# MAVS maintains mitochondrial import competence and also limits RNA virus replication independently of interferon signaling

**DOI:** 10.1128/mbio.02071-26

**Published:** 2026-09-29

**Authors:** Vishal Sah, Poojitha Sai Potharaju, Karthika S. Nair, Prangya Paramita Sahoo, Ravicanti Abhiram Pooja, Debasmita Basu, Dixit Tandel, K. S. Varadarajan, Santosh Chauhan, Anant Bahadur Patel, Krishnan Harinivas Harshan

**Affiliations:** 1 CSIR-Centre for Cellular and Molecular Biology 28569, Hyderabad, Telangana, India; 2 Academy for Scientific and Innovative Research (AcSIR), Ghaziabad, India; National Institutes of Health, Bethesda, Maryland, USA

**Keywords:** MAVS, protein import, RLR pathway, interferon-independent, mitophagy, mitochondrial dynamics, viral replication, host-pathogen interactions, SARS-CoV-2, JEV, DENV, RNA viruses

## Abstract

Intrinsic, cell-autonomous antiviral defenses can restrict infection without involving paracrine interferon (IFN) programs. Previous studies have demonstrated the importance of intact mitochondrial homeostasis in antiviral immunity. Mitochondrial antiviral signaling (MAVS) protein is a critical adaptor molecule in the RLR pathway that regulates IFN production in response to RNA viral infections. Here, we identify a potent intrinsic antiviral immunity that is orchestrated through stable mitochondria. We further identify MAVS as a regulator of mitochondrial import machinery whose activity is required for an IFN-independent antiviral state. Loss of MAVS leads to severe mitochondrial fragmentation, depolarization, and mitophagy, accompanied by diminished mitochondrial bioenergetics and protein import. Mechanistically, MAVS maintains mitochondrial integrity by maintaining the expression and assembly of the translocase of the outer membrane complex and sustaining the abundance of its core components. Restoration of MAVS expression reverses these defects and reinstates a robust antiviral state. Remarkably, this mitochondrial-driven immunity efficiently restricts SARS-CoV-2 replication even under IFN-deficient conditions and operates alongside the IFN pathways during infection by RNA viruses, such as the Japanese encephalitis virus. Our findings reveal a mitochondrial maintenance function of MAVS that operates in parallel to, and independently of, canonical interferon signaling during RNA virus infection. These findings highlight mitochondrial integrity as a common determinant behind a broad, collective antiviral immunity that involves both intrinsic and IFN-dependent mechanisms.

**IMPORTANCE:**

How do mitochondria contribute to the intrinsic cellular antiviral defense even when the primary immune arsenal is silenced? This work reveals that MAVS, long known for triggering interferon, acts as a vital bridge that stabilizes mitochondria to impart a potent intrinsic antiviral state against RNA viruses. We show that MAVS serves as a structural guardian of the organelle, a function it orchestrates by stabilizing the translocase of the outer membrane complex, the essential gateway for mitochondrial protein import. When MAVS is lost or targeted by viruses like SARS-CoV-2 and Japanese encephalitis virus (JEV), this gateway collapses, leading to organelle failure that viruses exploit. Crucially, we demonstrate that MAVS can restrict viral replication entirely independently of traditional interferon signaling. This discovery shifts our understanding of MAVS from a simple signal transducer to a multidimensional protector. By safeguarding the cell’s “powerhouse,” MAVS provides a fundamental layer of intrinsic immunity that remains active even when other immune responses are evaded.

## INTRODUCTION

Interferon (IFN)- and cytokine-mediated mechanisms dominate our understanding of innate antiviral immunity. However, cells also deploy intrinsic, cell-autonomous antiviral defenses which are pre-existing, rapidly acting mechanisms that can restrict infection immediately within the infected cell, often before a paracrine interferon program is established (1–3). In classical usage, intrinsic immunity is mediated by restriction factors that directly inhibit discrete steps of the viral life cycle (4, 5), whereas “innate immunity” is often framed as a linear cascade of pathogen sensing–signaling–IFN/cytokine induction that coordinates local-to-systemic responses. How these two layers of antiviral defense intersect within infected cells remains incompletely understood. RNA sensing by cytosolic RIG-I-like receptors (RIG-I and MDA5) provides a useful lens to examine this intersection. Upon detection of viral RNA, these receptors signal through the MAVS protein, anchored on mitochondria and peroxisomes (6, 7). MAVS activation involves CARD-dependent oligomerization into higher-order assemblies that drive type I and type III interferon production and interferon-stimulated gene (ISG) expression (8, 9). Because this signaling output is essential for canonical innate immunity, diverse viruses antagonize MAVS or its assembly to blunt interferon responses (10–14). As MAVS-dependent antiviral signaling is primarily coordinated from mitochondria, this places the organelle itself at the center of host–virus interactions during infection.

Mitochondria are central hubs for innate antiviral defense, coordinating RNA sensing, signal transduction, organelle quality control, and metabolic adaptation during infection (15, 16). Intact mitochondrial homeostasis supports IFN-dependent defenses. Many RNA viruses remodel mitochondrial architecture to create a permissive cellular state (17–19). Viral infection has been associated with mitochondrial fragmentation, fusion-biased elongation, loss of membrane potential, altered bioenergetics, and engagement of mitophagy (20, 21). These mitochondrial responses are highly dynamic and context-dependent, reflecting both virus-encoded activities and host attempts to maintain organellar homeostasis (17, 19, 22–26). Notably, many of these perturbations converge at the outer mitochondrial-associated membranes (MAMs) and mitochondrial–ER contact sites (MERCs), where antiviral signaling complexes assemble, and metabolic exchange is coordinated (27, 28). Despite extensive documentation of virus-induced mitochondrial remodeling, it remains unclear which host factors normally stabilize these mitochondrial territories during infection, how this buffering is lost, and whether preserving organellar integrity itself constitutes a barrier to viral replication.

One core function embedded within these same outer-membrane territories is mitochondrial protein import. It is a tightly regulated, multi-step process executed by translocases in the outer and inner membranes (TOM/TIM). Because mitochondrial fitness depends on continuous TOM-/TIM-mediated import of nuclear-encoded proteins (23, 24), disruption of import is expected to destabilize respiratory function and membrane potential and engage PINK1/Parkin-linked quality control, including mitophagy (25, 26). Of the many components of the TOM complex, the TOM receptors TOM20 and TOM70 form the precursor-recognition module at the outer membrane, a key interface between mitochondrial maintenance and antiviral signaling. Additionally, TOM70 has been reported to interact with MAVS during RNA virus infection and to facilitate recruitment of downstream TBK1/IRF3 signaling machinery to mitochondria (29). Consistent with its central role at the intersection of mitochondrial import and antiviral signaling, several RNA viruses directly target TOM70. While TOM70 has been shown to interact with MAVS to facilitate TBK1/IRF3 recruitment, whether MAVS reciprocally regulates TOM complex stability and mitochondrial import competence has not been examined (26, 30, 31). MAVS signaling itself is coordinated from MAMs, placing antiviral signalosome assembly within outer-membrane territories that also govern protein handling and organelle quality control. Together, these observations place the TOM receptor module, and TOM70 in particular, at a nexus where import competence, mitochondrial integrity, and antiviral signaling intersect.

While investigating mitochondrial dysfunction during SARS-CoV-2 (CoV-2) infection, we observed that MAVS status critically determines mitochondrial import competence through its regulation of TOM complex organization. Importantly, this antiviral activity persists under conditions of complete IFN pathway blockade, suggesting that the mitochondrial maintenance role of MAVS contributes to a second, IFN-independent layer of antiviral defense. We present evidence to establish that MAVS is required to maintain mitochondrial network integrity, membrane potential, and oxidative phosphorylation during viral infections and otherwise. Its loss destabilizes core import machinery, remodels TOM organization, and elevates PINK1-linked mitophagy. Restoring MAVS re-establishes TOM receptor organization, import competence, and organellar fitness, reinstating antiviral resistance. Notably, this intrinsic immunity is capable of potently restricting viruses not only under conditions of IFN deficiency as in SARS-CoV-2 infections, but can also operate in parallel with canonical IFN pathways during infections by viruses such as JEV, where IFN signaling is active. Together, our findings position MAVS as a mitochondrial guardian whose preservation of organelle integrity oversees an IFN-independent axis of antiviral defense, in addition to the canonical IFN-dependent programs.

## RESULTS

### MAVS is critical for maintaining mitochondrial structure and homeostasis

**Fig 1:**
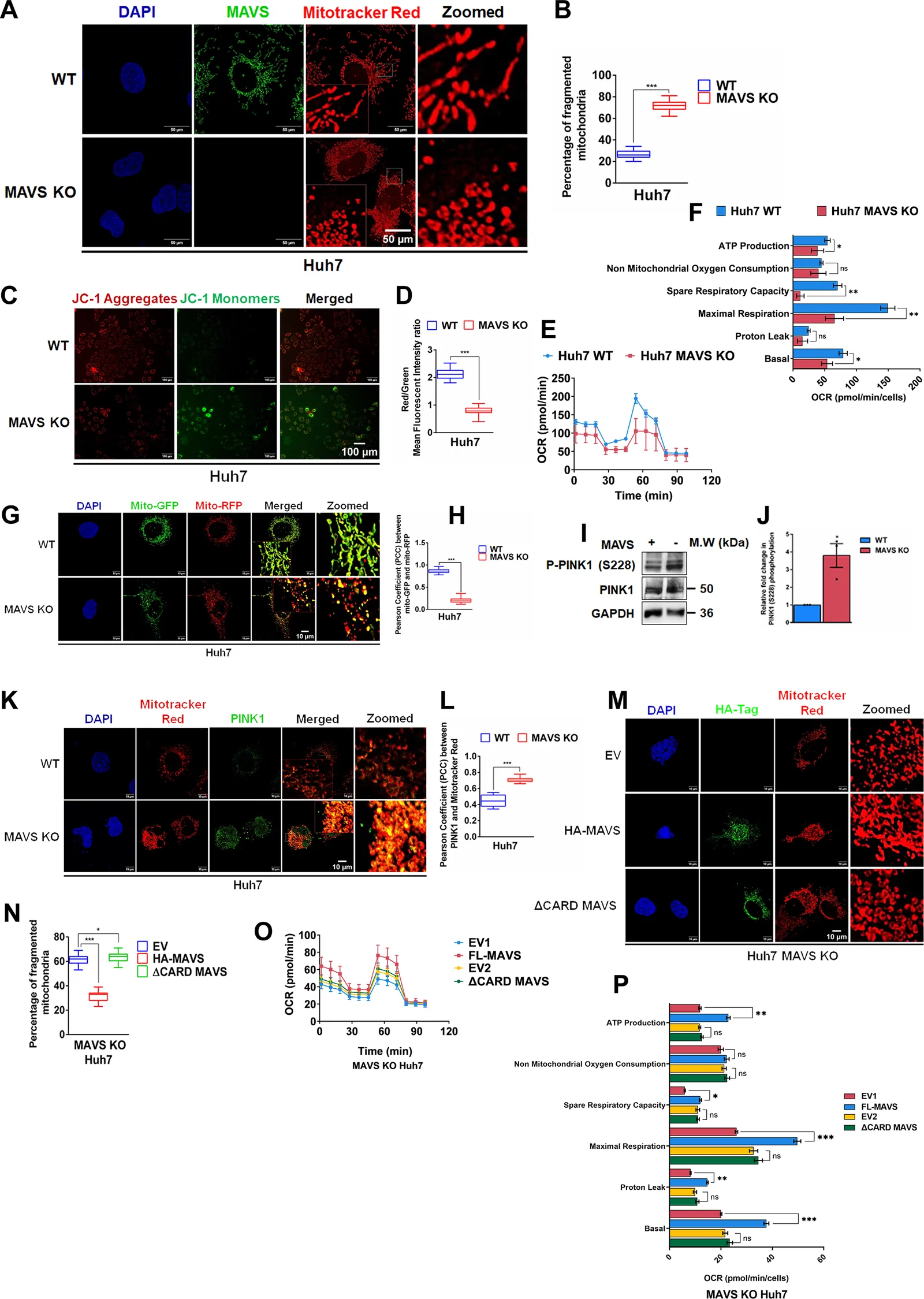
MAVS is critical for maintaining mitochondrial structure and homeostasis. (**A**) Confocal images showing mitochondrial fragmentation in Huh7 WT and MAVS KO cells, and (**B**) its corresponding quantification. Scale bars, 50 µm. (**C**) Epifluorescence microscopy images analyzing mitochondrial membrane potential in WT and MAVS KO Huh7 cells. Scale bars = 100 µm, and (**D**) quantitative representation of mitochondrial membrane potential from images in panel **C** (*n* = 100 cells). (**E**) OCR and (**F**) mitochondrial respiration parameters in WT and MAVS KO Huh7 cells measured by Mito Stress Assay and analyzed using the Seahorse XFe24 analyzer (*n* = 5). (**G**) Confocal images showing mitophagy in Huh7 WT and MAVS KO cells transfected with a Mito-GFP-RFP reporter and (**H**) corresponding quantification of mitophagy. (**I**) Immunoblot analysis of PINK1 phosphorylation in WT and MAVS KO cells and (**J**) densitometric quantification. (**K**) Confocal images showing co-localization of PINK1 (green) with mitochondria stained with Mitotracker Red (red) in WT and MAVS KO cells and (**L**) corresponding quantification of co-localization. (**M**) Confocal images showing mitochondrial fragmentation in MAVS KO Huh7 cells transfected with FL-MAVS or ΔCARD MAVS and (**N**) corresponding quantification. (**O**) OCR and (**P**) mitochondrial respiration parameters in MAVS KO cells transfected with FL-MAVS (matched to pUNO empty vector [EV]) or ΔCARD-MAVS (matched to pcDNA empty vector), measured by Mito Stress Assay (*n* = 5). Scale bars, 10 µm and *n* = 50 cells unless otherwise indicated. Data are presented as mean ± SEM, with *, **, and *** denoting *P* < 0.05, 0.01, and <0.001, respectively.

MAVS loss decapitates the RLR signaling, resulting in a muted IFN response upon viral infection (6, 12). In MAVS knockout (KO) Huh7 cells (generated using CRISPR/Cas9), we interestingly noticed that mitochondria were heavily fragmented as compared against their wild-type (WT) counterparts (puromycin selected post-vector transfection), which maintained normal mitochondrial population (Fig. 1A and B). Similar changes were noticed in multiple individual KO clones (Fig. S1A) and also in primary mouse embryonic fibroblasts (MEFs) from Mavs^−/−^ mice (Fig. S1B through D), suggesting that this association of MAVS with mitochondrial morphology is well conserved across organisms. Given this significant morphological alteration, we next assessed mitochondrial membrane potential (Δψm). JC-1 staining (a ratiometric dye whose red/green fluorescence ratio reports mitochondrial membrane potential) revealed a significant reduction in Δψm in MAVS KO Huh7 and Mavs^−/−^ MEFs relative to their WT controls (Fig. 1C and D; Fig. S1E and F). Because electron transport chain activity and Δψm are tightly coupled (32), we profiled mitochondrial bioenergetics using a Seahorse XF analyzer. MAVS-KO Huh7 cells showed significantly reduced basal/maximal oxygen consumption rate (OCR), collapsed spare capacity, and lower ATP-linked respiration (Fig. S1G; Fig. 1E and F). Similar observations were made in primary Mavs^−/−^ MEFs (Fig. S1H and I). As a consequence of these changes, MAVS KO cells displayed markedly slower growth rates (Fig. S1J). Failure to sustain efficient mitochondrial energetics in MAVS-KO cells prompted us to ask whether this bioenergetic collapse engages mitochondrial quality control pathways. MitoGFP-RFP flux assay reported increased RFP-only (acidified) puncta per cell, indicating elevated mitophagy flux in MAVS KO Huh7 and Mavs^−/−^ MEFs (Fig. 1G and H; Fig. S1K and L). Elevated PINK1 phosphorylation and its colocalization with mitochondria (Fig. 1I through L) indicated activated PINK1-dependent mitophagy in MAVS-deficient cells.

To confirm the association of MAVS with these changes, we re-expressed HA-MAVS in MAVS-KO Huh7 cells and reassessed mitochondrial integrity. Full-length MAVS restored tubular mitochondrial networks (Fig. 1M and N) in MAVS KO Huh7 cells. As a loss-of-oligomerization control, we introduced a CARD-deleted mutant (ΔCARD-MAVS), an established approach to prevent productive MAVS aggregation while preserving mitochondrial localization (8, 33). ΔCARD-MAVS failed to restore mitochondrial networks. Across all Seahorse parameters such as basal respiration, maximal respiration, ATP production, spare respiratory capacity, and proton leak, FL-MAVS produced a significant increase over its matched empty vector control (pUNO), while ΔCARD-MAVS showed no significant change relative to its matched control (pcDNA) in MAVS KO cells (Fig. 1O and P). In MAVS-competent cells, full-length MAVS supplementation similarly enhanced mitochondrial network integrity and bioenergetics, while ΔCARD-MAVS had no effect (Fig. S1M through P). These data show that MAVS is necessary to maintain mitochondrial homeostasis.

### MAVS safeguards mitochondria by maintaining TOM complex expression and protein import competence

**Fig 2:**
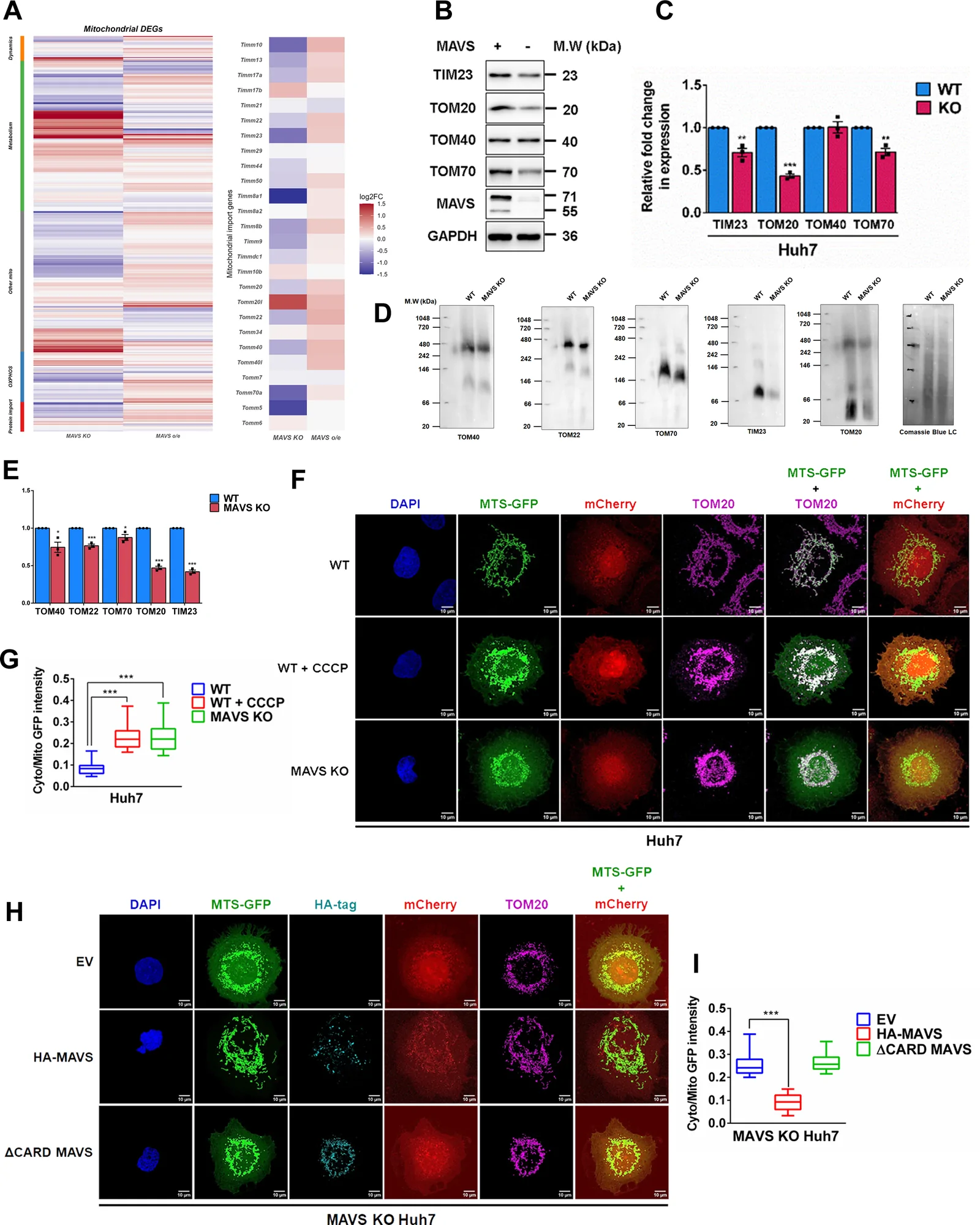
MAVS safeguards mitochondria by maintaining TOM complex expression and protein import competence. (**A**) Heatmap showing the most significantly regulated mitochondrial DEGs, and the DEG subset of genes responsible for mitochondrial protein import. MAVS-OE Huh7 cells and MAVS-KO MEFs were each analyzed independently against their own species-matched controls (MAVS-OE Huh7 vs empty-vector Huh7; MAVS-KO MEF vs WT MEF) in separate DESeq2 runs. Human and mouse DEG sets were subsequently aligned by orthologous gene identity via homolog mapping for joint visualization. Expression values are represented as log_2_ fold change. (**B**) Immunoblot analysis of TIM23, TOM20, TOM40, TOM70, and MAVS levels in Huh7 WT and MAVS KO cells and (**C**) the corresponding quantification of their relative levels. (**D**) BN-PAGE analysis of TOM40, TOM22, TOM70, TIM23, and TOM20 abundance in enriched mitochondria prepared from Huh7 WT and MAVS KO cells and (**E**) corresponding quantification. (**F**) Confocal images showing cytosolic accumulation of MTS-GFP in Huh7 WT and MAVS KO cells and (**G**) corresponding quantification of the Cyto/Mito ratio. CCCP treatment was used as a positive control. (**H**) Confocal images showing cytosolic accumulation of GFP in Huh7 MAVS KO cells supplemented with FL-MAVS and ∆CARD MAVS and (**I**) corresponding quantification of the Cyto/Mito ratio. Scale bars, 10 µm, and *n* = 50 cells unless otherwise indicated. Data are presented as mean ± SEM, with *, **, and *** denoting *P* < 0.05, 0.01, and <0.001, respectively.

The magnitude of mitochondrial defects upon MAVS perturbation suggested broad remodeling of mitochondrial programs. To define mitochondrial programs responsive to MAVS, we carried out deep RNA sequencing in MAVS-overexpressing Huh7 cells and in primary Mavs^−/−^ MEFs. Gene Ontology (GO) enrichment analysis of upregulated and downregulated differentially expressed genes (DEGs) across both systems revealed antiviral and IFN-response categories alongside prominent mitochondrial structural, metabolic, and inner membrane complex terms, confirming that MAVS regulates a transcriptional program extending beyond canonical immune gene regulation (Fig. S2A through D). Using these perturbations across different animal models, we tested whether shared mitochondrial pathways were consistently impacted by doing a cross-species comparison. MAVS-OE Huh7 cells and MAVS-KO MEFs were each analyzed against their own species-matched controls in separate DESeq2 runs, and the resulting human and mouse DEG sets were aligned by homolog mapping for joint visualization. Mitochondrial enrichment analysis showed a strong correlation between MAVS and mitochondrial protein import. Several components of the translocase of the outer mitochondrial complex were downregulated in MAVS KO MEFs and upregulated in MAVS overexpressing Huh7 cells (Fig. 2A; Fig. S2E). Their positive correlation with MAVS expression strongly suggested that MAVS is critical for the TOM complex assembly. To exclude the possibility that upregulation of import machinery mRNAs reflects indirect transcriptional activation by IRF3 or STAT1 downstream of MAVS oligomerization, we queried the ENCODE and ChEA Consensus ChIP-seq data sets via Enrichr (34, 35). Neither IRF3 nor STAT1 was significantly enriched as a transcriptional regulator of TIMM or TOMM genes (Fig. S2F and G). These observations were further confirmed by immunoblotting (Fig. 2B and C). TOM20 and TOM70, two important accessory components of the TOM complex, were significantly underexpressed in MAVS KO cells, indicating selective loss of core import components. In contrast, TOM40, which is the central β-barrel protein-conducting channel through which all translocating preproteins physically pass, was not significantly regulated, suggesting that MAVS selectively maintains the receptor-facing components of the TOM complex rather than its structural pore. Additionally, TIM23, a key component of the translocase of the inner mitochondrial TIM23 complex, also showed compromised expression in these cells. Importantly, this regulation occurs at the transcript level, as evidenced by the concordant RNA-seq changes preceding the protein-level alterations. Blue native PAGE (BN-PAGE) analysis of mitochondrion-enriched fractions showed reduced assemblies of TOM core and accessory components (Fig. 2D and E). TIM23-containing assemblies were similarly reduced/redistributed, indicating compromised TIM23 complex integrity. Because TOM70 has been reported to interact with MAVS (29) and TOM20 associates with TOM70 within the TOM receptor module (36), we asked whether MAVS loss disrupts TOM20/TOM70 organization on the mitochondrial surface. High-resolution immunostaining with object-based quantification showed reduced TOM20 and TOM70 puncta densities (~40% each) after normalization to mitochondrial area (Fig. S2H through J). Object-based colocalization further revealed ~50% fewer TOM20–TOM70 intersections and increased median centroid-to-centroid distances (Fig. S2K through M), consistent with reduced TOM receptor clustering in the absence of MAVS. Since a major disorganization of the TOM complex is likely to impact mitochondrial protein import, we assessed it directly using a mitochondrial targeting signal (MTS)-GFP reporter. MAVS-KO Huh7 cells displayed increased accumulation of cytosolic GFP, comparable to treatment of WT cells with carbonyl cyanide m-chlorophenylhydrazone (CCCP; a common mitotoxin) (Fig. 2F and G), consistent with impaired mitochondrial import. This import defect was rescued by MAVS re-expression: HA-MAVS restored mitochondrial targeting of the MTS-GFP reporter and reduced cytosolic GFP accumulation in MAVS-KO Huh7 cells, whereas the ΔCARD mutant failed to restore import (Fig. 2H and I). This import defect is consistent with, and likely contributes to, the PINK1 accumulation and elevated mitophagy observed in MAVS-KO cells, given that PINK1 stabilizes on dysfunctional, import-incompetent mitochondria. Together, these data show that MAVS preserves TOM receptor abundance and organization to sustain mitochondrial import competence.

### SARS-CoV-2 prevents MAVS aggregation coincident with mitochondrial destabilization

**Fig 3:**
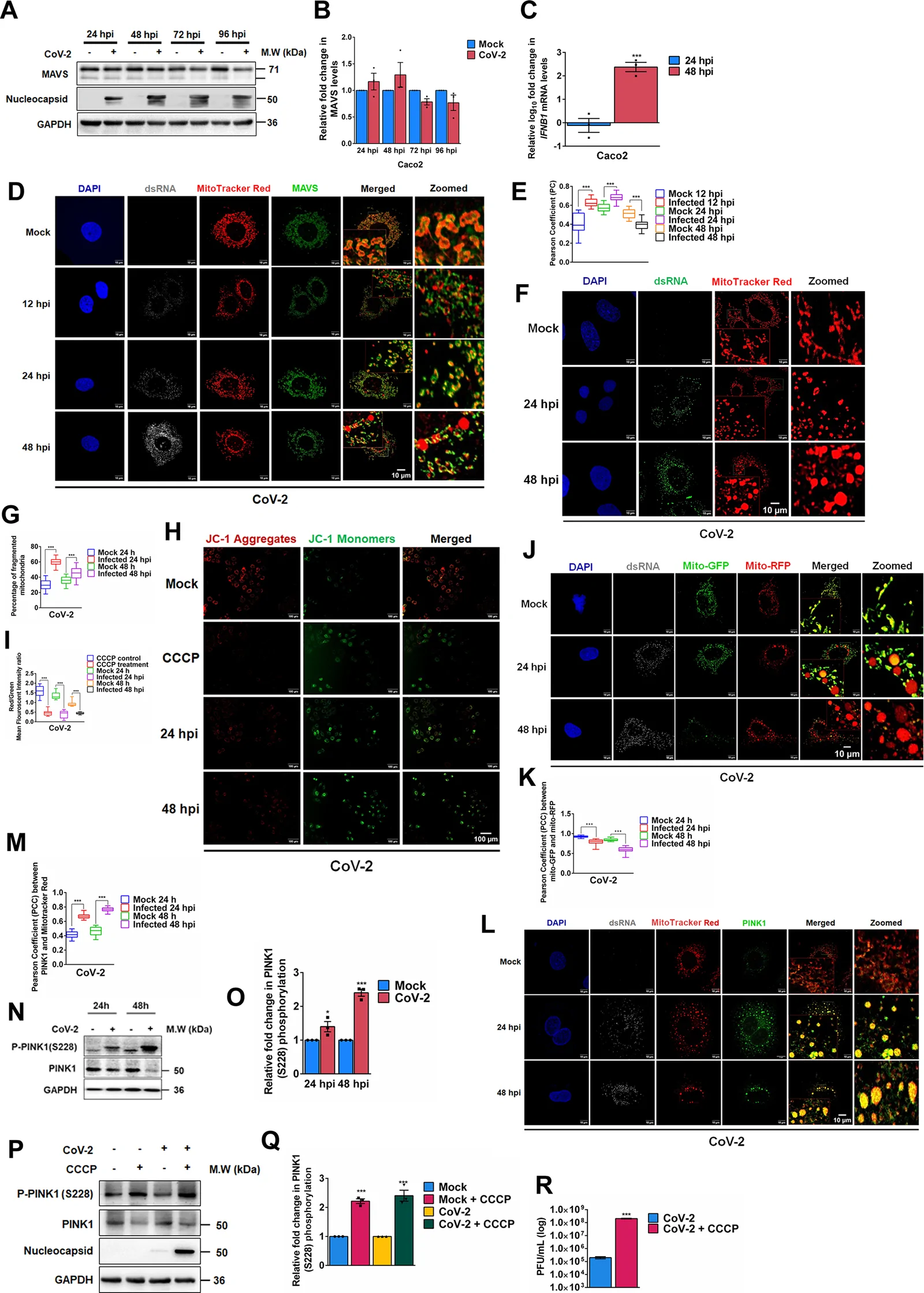
SARS-CoV-2 prevents MAVS aggregation coincident with mitochondrial destabilization. (**A**) Immunoblot analysis of MAVS and SARS-CoV-2 nucleocapsid protein expression levels shown at different time points upon SARS-CoV-2 infection in Caco-2 cells and (**B**) its densitometric analysis. (**C**) Real-time quantitative RT-PCR (qRT-PCR) analysis showing relative fold change in *IFNB1* levels in Caco-2 cells at 24 and 48 hpi. (**D**) Confocal images showing MAVS aggregation in Huh7 cells infected with SARS-CoV-2, stained with Mitotracker Red and immunostained for dsRNA (dark gray) and MAVS (green). (**E**) Quantification of Pearson’s correlation coefficient (PCC) for MAVS-mitochondrial co-localization from panel **D** (*n* = 250 ROIs). (**F**) Confocal images showing mitochondrial fragmentation in SARS-CoV-2-infected Huh7 cells. (**G**) Quantification of mitochondrial fragmentation from panel **F**. (**H**) Epifluorescence microscopy images (10×) analyzing mitochondrial membrane potential through JC1 staining at different time points post- SARS-CoV-2 infection, with CCCP as a positive control. (**I**) Quantification of mitochondrial membrane potential from panel **H** (*n* = 100 cells). Scale bars, 100 µm. (**J**) Confocal images showing mitophagy in SARS-CoV-2-infected Huh7 cells transfected with a Mito-GFP-RFP reporter and (**K**) corresponding quantification of mitophagy. (**L**) Confocal images showing PINK1 (green) co-localization with MitoTracker Red (red) in infected cells and (**M**) quantification of PINK1 localization. (**N**) Immunoblot analyzing PINK1 phosphorylation (S228) in SARS-CoV-2-infected Huh7 cells and (**O**) densitometric quantification. (**P**) Immunoblot analyzing PINK1 phosphorylation following CCCP treatment in uninfected or SARS-CoV-2-infected Huh7 cells for 24 h. (**Q**) Densitometric analysis of PINK1 phosphorylation from panel **P**. (**R**) Infectious viral titers from corresponding supernatants. Scale bars, 10 µm and *n* = 50 cells unless otherwise indicated. Data are presented as mean ± SEM, with *, **, and *** denoting *P* < 0.05, 0.01, and <0.001, respectively.

RNA viruses are known to target MAVS to blunt innate immune signaling (11, 13, 37, 38). Given that MAVS loss resulted in mitochondrial destabilization, we hypothesized that loss of MAVS oligomerization is associated with mitochondrial defects that mirror MAVS deficiency. We and others have previously reported that SARS-CoV-2 dampens IRF3 activation and IFN signaling (10, 39–41). Consistent with this, SARS-CoV-2 failed to alter MAVS abundance (Fig. 3A and B; Fig. S3A and B) or to induce a type I interferon response (Fig. 3C; Fig. S3C) (10). In contrast, and consistent with prior reports (42, 43) and our previous work (44), JEV infection robustly activated RLR–IRF3 signaling by 24 h post-infection (hpi) (Fig. S3D), confirming engagement of the MAVS pathway under these conditions. We therefore tested whether SARS-CoV-2 perturbs MAVS oligomerization.

MAVS aggregation was quantified using a confocal colocalization-based assay previously described (45), with aggregation scored by the Pearson coefficient between MAVS and mitochondrial signals (Fig. S3E). In this assay, MAVS aggregation is reflected by a decrease in PCC: condensation of MAVS into discrete puncta on a subset of mitochondria reduces pixel-level correlation with the bulk mitochondrial signal and gives a low PCC, whereas diffuse, non-aggregated MAVS yields a high PCC (45). Poly(I:C) stimulation robustly increased MAVS aggregation (low PCC), whereas the non-oligomerizing ΔCARD-MAVS mutant, which gave a higher PCC, served as a negative control (Fig. S3F and G) (13, 46, 47). SARS-CoV-2 infection failed to increase MAVS aggregation across the infection time course (Fig. 3D and E). Notably, mitochondria in dsRNA-positive SARS-CoV-2-infected cells were largely devoid of MAVS puncta, indicating exclusion of MAVS from the mitochondrial network during infection (Fig. S3H). We next asked whether this redistribution of MAVS during SARS-CoV-2 infection is accompanied by changes in mitochondrial organization. SARS-CoV-2 infection induced extensive mitochondrial fragmentation (Fig. 3F and G), loss of mitochondrial membrane potential (Fig. 3H and I), and increased mitophagosome formation (Fig. 3J and K), accompanied by increased PINK1 accumulation and phosphorylation (Fig. 3L through O). JEV and dengue virus (DENV) have previously been shown to target MAVS and its aggregation (47, 48). Similar to SARS-CoV-2, JEV induced mitochondrial fragmentation and loss of membrane potential (Fig. S3I through L), accompanied by elevated mitophagosome formation (Fig. S3M and N), whereas DENV promoted mitochondrial elongation while largely preserving mitochondrial homeostasis, consistent with previous reports (22–24). These differences suggested a pro-viral role for mitophagy, prompting us to investigate this association. Interestingly, CCCP-induced mitophagy significantly enhanced SARS-CoV-2 replication (Fig. 3P through R; Fig. S3O and P), and JEV showed a similar increase (Fig. S3Q and R). In contrast, CCCP treatment reduced DENV infection (Fig. S3S and T), indicating that mitophagy promotes SARS-CoV-2 and JEV infection but is not beneficial and may be detrimental to DENV under these conditions. These results indicated that failure of MAVS to undergo productive aggregation during SARS-CoV-2 infection coincides with the mitochondrial dysfunction that characterizes infection, consistent with a model in which viral inhibition of MAVS assembly contributes to mitochondrial destabilization. It is likely that DENV coordinates unique mechanisms to counter the mitophagy induction resulting from MAVS disaggregation.

### MAVS implements mitochondrial homeostasis to restrict RNA virus replication

**Fig 4:**
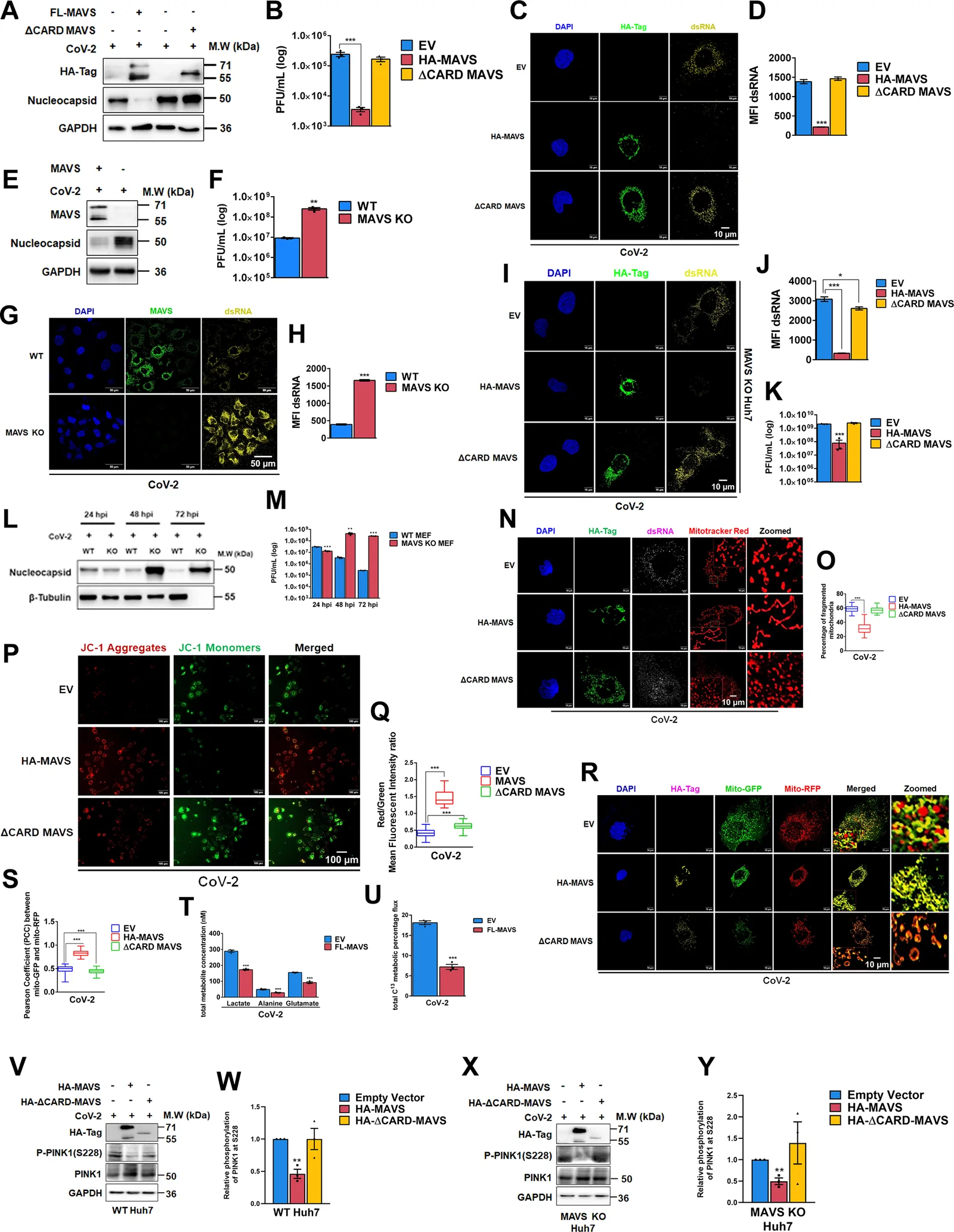
MAVS implements mitochondrial homeostasis to restrict RNA virus replication. (**A**) Immunoblot showing HA-MAVS and SARS-CoV-2 nucleocapsid levels in Huh7 cells overexpressing HA-tagged FL-MAVS or ΔCARD MAVS after SARS-CoV-2 infection. Full-length HA-MAVS migrates at ~71 kDa (calculated ~57 kDa; anomalous migration due to post-translational modifications); the ~55 kDa band is mini-MAVS (Met142 internal-initiation isoform, HA-tagged). ΔCARD-MAVS migrates at ~66 kDa, below full length. (**B**) Infectious viral titers from corresponding supernatants from panel **A**. (**C**) Confocal images detecting dsRNA in similarly treated cells and (**D**) quantification of dsRNA mean fluorescence intensity. (**E**) Immunoblot showing MAVS and SARS-CoV-2 nucleocapsid levels in WT and MAVS KO Huh7 cells following infection and (**F**) corresponding infectious viral titers. (**G**) Confocal images of dsRNA staining and MAVS expression in MAVS KO Huh7 and their WT cells and (**H**) quantification of dsRNA intensity in samples from panel **G** (*n* = 100 cells). (**I**) Confocal images showing dsRNA in MAVS KO Huh7 cells transfected with HA-tagged FL-MAVS or ΔCARD MAVS and infected with SARS-CoV-2 and (**J**) dsRNA intensity quantification (*n* = 100 cells). (**K**) Infectious titers from corresponding supernatants. (**L**) Immunoblot analyzing SARS-CoV-2 nucleocapsid levels in WT and MAVS KO MEFs infected with SARS-CoV-2 at different time points. (**M**) Infectious viral titers comparing infection in WT and MAVS KO MEFs from panel **L**. (**N**) Confocal images analyzing mitochondrial fragmentation in SARS-CoV-2-infected Huh7 cells overexpressing HA-tagged FL-MAVS or ΔCARD-MAVS and (**O**) corresponding quantification. (**P**) Epifluorescence microscopy images (10×) analyzing mitochondrial membrane potential in SARS-CoV-2-infected Huh7 cells upon supplementation with FL-MAVS or ΔCARD-MAVS. Scale bars = 100 µm, and (**Q**) Quantitative representation of mitochondrial membrane potential from images in panel **P** (*n* = 100 cells). (**R**) Confocal images analyzing mitophagy using the mito-GFP-RFP assay in similarly treated cells as in panel **Q** and (**S**) corresponding quantification. (**T**) Quantification of total glycolytic metabolite levels and (**U**) ATP synthesis rates by 1D metabolic NMR in SARS-CoV-2-infected Huh7 cells overexpressing FL-MAVS. (**V**) Immunoblot analysis of PINK1 phosphorylation in SARS-CoV-2-infected Huh7 WT cells overexpressing FL-MAVS or ΔCARD-MAVS and (**W**) densitometric quantification. (**X**) Immunoblot analysis of PINK1 phosphorylation in SARS-CoV-2-infected MAVS KO Huh7 cells overexpressing FL-MAVS or ΔCARD-MAVS and (**Y**) corresponding quantification. Scale bars, 10 µm, and *n* = 50 cells unless otherwise indicated. Data are presented as mean ± SEM, with *, **, and *** denoting *P* < 0.05, 0.01, and <0.001, respectively.

Given that MAVS is a well-established antiviral adaptor, our finding of a mitoprotective role of MAVS suggested that MAVS is the link between mitochondrial homeostasis and antiviral immunity. We tested whether boosting MAVS can restore mitochondrial integrity during infection and thereby limit viral replication by observing MAVS gain- and loss-of-function in parallel across infection models. Full-length MAVS supplementation, but not the ΔCARD mutant, significantly reduced SARS-CoV-2 replication across multiple cell lines and experimental systems (Fig. 4A through D; Fig. S4A and B). A transmembrane domain-deleted MAVS mutant (ΔTM-MAVS/Cyto-MAVS), which mislocalizes diffusely to the cytosol, similarly failed to restrict SARS-CoV-2 replication (Fig. S4C), demonstrating that outer mitochondrial membrane (OMM) localization is required for antiviral activity independently of CARD-mediated oligomerization. Consistent with a MAVS-dependent restriction, MAVS-KO cells supported markedly higher SARS-CoV-2 titers than WT controls (Fig. 4E through H). Reintroduction of full-length MAVS into the KO background restored antiviral control, reducing viral titers across multiple cellular systems (Fig. 4I through K; Fig. S4D and E). Notably, loss of MAVS also enhanced viral replication in primary MEFs and A549 cells—two systems with relatively low ACE2 expression (Fig. S4F) and limited baseline permissivity to SARS-CoV-2 (49, 50)—to levels comparable to those observed in highly permissive models (Fig. 4L and M; Fig. S4G and H). Collectively, these results suggest that MAVS is a major determinant of SARS-CoV-2 tropism and possibly a broader viral permissivity.

Given MAVS’s role in mitochondrial homeostasis, we asked whether MAVS-mediated restriction preserves mitochondrial network organization during infection. Full-length MAVS, but not ΔCARD-MAVS, preserved mitochondrial network organization during SARS-CoV-2 infection, reducing infection-associated fragmentation (Fig. 4N and O). This morphological rescue reflects a direct mitochondrial function of MAVS rather than an indirect consequence of reduced viral burden: MAVS overexpression promotes mitochondrial elongation in mock-infected MAVS-KO and WT cells and in MAVS/IFNAR1 DKO cells in the complete absence of infection (Fig. 1M and N; Fig. S1M and N; and see Fig. 6E and F), demonstrating that this protective activity is independent of any viral context. Full-length MAVS, but not ΔCARD-MAVS, restored mitochondrial membrane potential and reduced infection-associated mitophagosome formation in SARS-CoV-2-infected cells (Fig. 4P through S). MAVS supplementation produced similar rescue effects during JEV infection but not DENV infection, consistent with the limited mitophagy engagement in DENV infection (Fig. S4I through L). Consistent with its role in maintaining mitochondrial integrity, MAVS supplementation in SARS-CoV-2 infection reduced glycolytic metabolite abundance and corresponding ATP synthesis rates relative to mock-infected cells (Fig. 4T and U; Fig. S4M and N), consistent with a viral Warburg-like metabolic shift (51, 52) that reflects mitochondrial dysfunction. SARS-CoV-2 dramatically upregulates glycolysis as mitochondrial function collapses; MAVS supplementation reduces this glycolytic surge. Together with the OCR rescue data (Fig. 1O and P), these observations suggest MAVS restores the metabolic balance between oxidative and glycolytic ATP production rather than simply increasing total energy output. FL-MAVS, but not ΔCARD-MAVS supplementation, reduced infection-induced PINK1 accumulation in both WT and MAVS-KO cells across multiple systems (Fig. 4V through Y; Fig. S4O through Q). Together, these data establish MAVS as a central mitochondrial safeguard that restricts viral replication by maintaining mitochondrial integrity and suppressing aberrant PINK1-dependent mitophagy during infection.

### MAVS restricts viral infection through an interferon-independent intrinsic antiviral program

**Fig 5:**
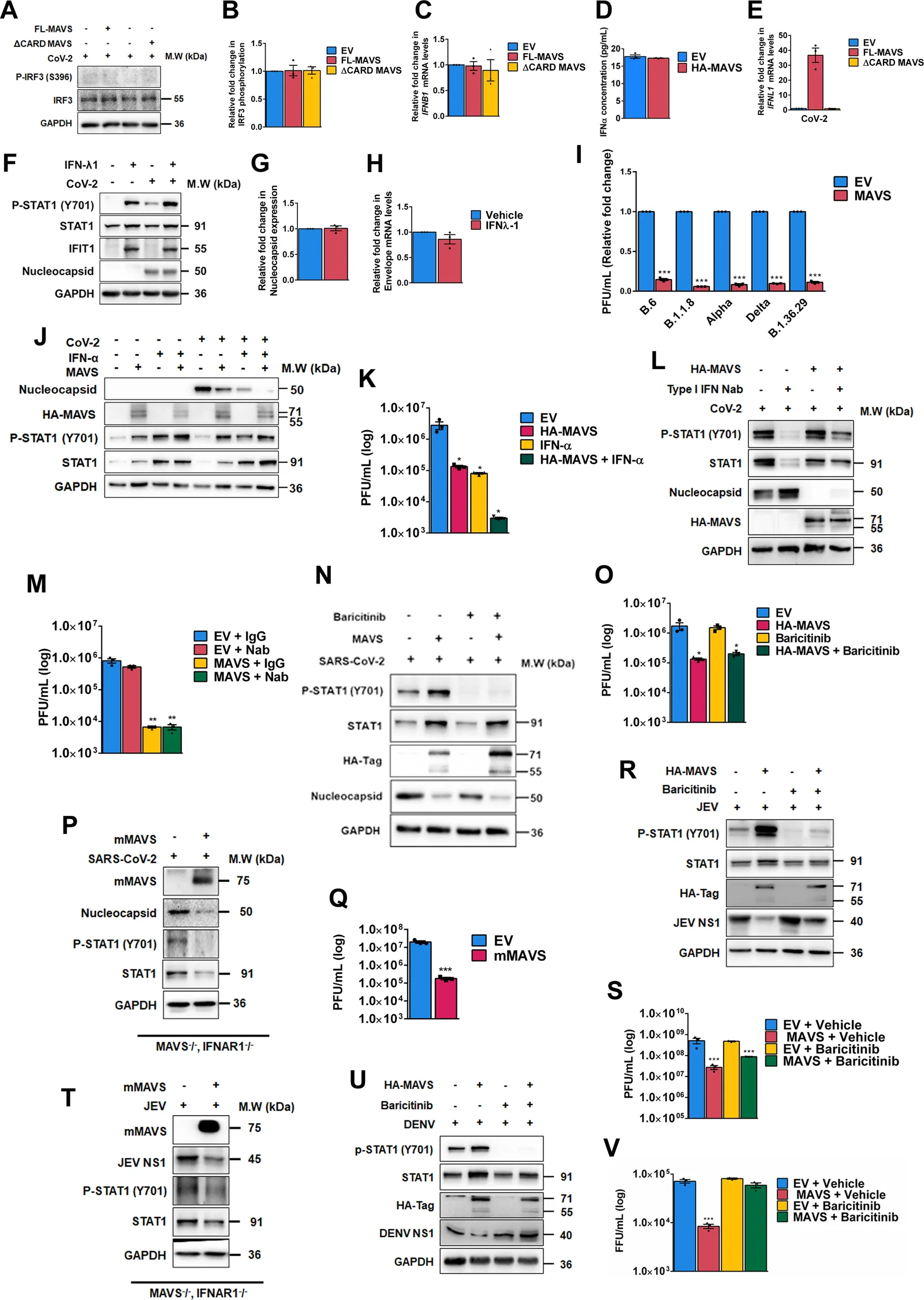
MAVS restricts viral infection through an interferon-independent intrinsic antiviral program. (**A**) Immunoblot analyzing IRF3 phosphorylation in SARS-CoV-2-infected Huh7 cells overexpressing FL-MAVS or ΔCARD MAVS. (**B**) Densitometric analysis of IRF3 phosphorylation. (**C**) qRT-PCR analysis of *IFNB1* mRNA levels for EV, FL-MAVS, and ΔCARD-MAVS conditions and (**D**) ELISA for IFN-α from their corresponding supernatants. (**E**) qRT-PCR analysis of *IFNL1* mRNA levels from panel **A**. (**F**) Immunoblot showing STAT1 phosphorylation, IFIT1, and SARS-CoV-2 nucleocapsid levels in IFN-λ-treated, SARS-CoV-2-infected Caco-2 cells and (**G**) nucleocapsid expression levels. (**H**) qRT-PCR of SARS-CoV-2 RNA from panel **F**. (**I**) Relative fold change in infectious viral titers upon MAVS overexpression in Huh7 cells infected with different SARS-CoV-2 variants. (**J**) Immunoblot analyzing STAT1 phosphorylation, SARS-CoV-2 nucleocapsid, and HA-MAVS levels in SARS-CoV-2-infected Huh7 cells overexpressing MAVS, treated with IFN-α or both and (**K**) infectious viral titers from corresponding supernatants. (**L**) Immunoblot showing STAT1 phosphorylation, HA-MAVS, and nucleocapsid levels in Huh7 cells treated with type I IFN neutralizing antibodies and (**M**) viral titers from their supernatants. (**N**) Immunoblot showing STAT1 phosphorylation, HA-MAVS, and nucleocapsid levels in Huh7 cells overexpressing MAVS, treated with baricitinib or their combination and (**O**) corresponding viral titers from their supernatants. (**P**) Immunoblot showing mMAVS, nucleocapsid expression, and STAT1 phosphorylation in SARS-CoV-2-infected MAVS:IFNAR DKO MEFs and (**Q**) infectious viral titers. (**R**) Immunoblot analysis of STAT1 phosphorylation, HA-MAVS, and JEV NS1 expression in Huh7 cells overexpressing HA-MAVS or treated with baricitinib or their combination, upon infection with JEV and (**S**) infectious viral titers from their supernatants. (**T**) Immunoblot analysis showing mMAVS, JEV NS1 expression, and STAT1 phosphorylation in MAVS:IFNAR DKO MEFs overexpressing mMAVS upon JEV infection. (**U**) Immunoblot analysis of STAT1 phosphorylation, HA-MAVS, and DENV NS1 expression in Huh7 cells overexpressing HA-MAVS, or treated with baricitinib or their combination, upon infection with DENV and (**V**) foci formation assay quantification of infectious viral titers from their supernatants. For studies involving transfection and infection, cells were transfected with 2 µg of each construct, infected with SARS-CoV-2 or JEV (MOI = 1) 24 h post-transfection (hpt), and stained or harvested 24 h post-infection. Data are presented as mean ± SEM, with *, **, and *** denoting *P* < 0.05, 0.01, and <0.001, respectively.

We noticed with surprise that despite causing potent SARS-CoV-2 suppression, MAVS supplementation during SARS-CoV-2 infection did not enhance IRF3 phosphorylation (Fig. 5A and B; Fig. S5A and B) nor induce *IFNB1* transcripts or secreted type I IFN (Fig. 5C and D; Fig. S5C). Importantly, MAVS robustly activated IRF3 phosphorylation and *IFNB1* expression and type I IFN in uninfected cells (Fig. S5D through G), and poly(I:C) transfection similarly induced robust *IFNB1* expression (Fig. S5H), together confirming intact interferon signaling competence in these cells. As a comparator, MAVS supplementation reduced JEV and DENV replication while enhancing type I IFN signaling (Fig. S5I through N), consistent with canonical RLR-IRF3-IFN antiviral signaling in these IFN-inducing infections (48, 53, 54). By contrast, MAVS restricted SARS-CoV-2 replication without detectable IFN amplification, indicating a distinct interferon-independent MAVS activity in this context. Because peroxisomal MAVS can also drive type III interferon responses (7), we examined IFN-λ induction following MAVS supplementation. MAVS robustly induced *IFNL1* expression in both SARS-CoV-2-infected and -uninfected cells (Fig. 5E; Fig. S5O). However, exogenous IFN-λ treatment failed to suppress SARS-CoV-2 replication (Fig. 5F through H), arguing against type III IFN as the primary mediator of MAVS-dependent restriction in this context. RNA-seq showed that MAVS supplementation elicits a transcriptional program distinct from IFN treatment, most prominently during SARS-CoV-2 infection (Fig. S5P), consistent with an IFN-independent antiviral state. Overlap analysis showed that MAVS and IFN share a comparable core ISG module in both mock and SARS-CoV-2 infection (~63% overlap), while each condition also induced a distinct set of additional ISGs (Fig. S5Q and R). Consistently, heatmaps revealed distinct MAVS- versus IFN-driven ISG signatures with a limited shared core (Fig. S5S and T), supporting the possibility of involvement of a MAVS-driven antiviral program outside the canonical IFN-mediated response during SARS-CoV-2 infection.

We have used an earlier variant isolate of SARS-CoV-2 throughout this study. Previous studies from our lab had shown that the earlier variants of SARS-CoV-2 show sensitivity to IFN-mediated antiviral defense, whereas later variants including alpha and delta showed remarkable resistance to it (10). Consistent with its proposed IFN-independent activity, MAVS supplementation also restricted replication of several SARS-CoV-2 variants, including alpha and delta (Fig. 5I). This indicated that MAVS-mediated restriction extends across SARS-CoV-2 variants that retain enhanced IFN evasion capabilities. MAVS supplementation and IFN-α treatment individually suppressed SARS-CoV-2 replication to comparable magnitudes. In support of MAVS’ IFN-independent action, their combination generated a more profound reduction in viral titers (Fig. 5J and K), indicating that they operated through parallel antiviral pathways. If MAVS and IFN-α operated through the same antiviral pathway, their combination would be expected to produce no greater effect than either alone, which is a hallmark of pathway redundancy. The substantially greater reduction in viral titers observed with the combination indicates that MAVS and IFN-α engage distinct, non-redundant mechanisms, providing independent evidence for the IFN independence of MAVS-mediated restriction. Further supporting these observations, MAVS-mediated suppression of SARS-CoV-2 replication prevailed unperturbed even under type I IFN receptor neutralization (Fig. 5L and M), and pharmacological inhibition of JAK-STAT signaling by baricitinib (Fig. 5N and O). To definitively establish whether MAVS-mediated antiviral restriction operates independently of type I IFN signaling, we employed primary MEFs from MAVS/IFNAR1 double-knockout mice—cells that are genetically incapable of both MAVS-dependent IFN induction and IFN receptor-mediated signaling. We generated MAVS/IFNAR1 double-knockout mice and tested MAVS’s antiviral potential in primary MEFs generated from them. MAVS supplementation significantly suppressed SARS-CoV-2 replication (Fig. 5P and Q), confirming the interferon-independent MAVS antiviral mechanism in a genetic background. Importantly, in this MAVS-IFNAR1-null context, MAVS reconstitution did not elevate total STAT1 levels relative to the empty vector condition (Fig. 5P) in contrast to the upregulation observed in MAVS-overexpressing WT Huh-7 cells (Fig. 5J, L, and N). Because this effect is lost when IFNAR1 is genetically absent, MAVS-driven STAT1 induction is IFNAR-dependent, arguing against a MAVS-proximal, IFN-independent transcription factor such as IRF3 as its driver (Fig. S2F and G). Because MAVS can also activate NF-κB (6), we inhibited NF-κB signaling with BAY11-7082 and found unaltered antiviral activity upon MAVS supplementation (Fig. S5U and V), indicating that MAVS-mediated SARS-CoV-2 restriction is also NF-κB-independent. Similarly, when interferon signaling was blocked pharmacologically or genetically, MAVS supplementation continued to suppress JEV replication (Fig. 5R through T). This result suggested that the IFN-independent viral restriction operates in parallel to the IFN-mediated mechanisms, and that it can continue to operate even upon IFN suppression. In contrast, MAVS-mediated suppression of DENV reverted upon interferon blockade (Fig. 5U and V), indicating that the IFN-independent antiviral defense operating upon SARS-CoV-2 and JEV infections does not operate during DENV infection. This is consistent with the clear absence of mitophagy during DENV infection and the lack of impact of mitophagy on its replication. Collectively, these data establish that MAVS elicits an antiviral response that is not restricted to IFN- and NF-κB signaling outputs but also by invoking a very potent intrinsic alternative mechanism that involves mitochondrial stabilization.

### MAVS maintains mitochondrial integrity independently of IFN signaling

**Fig 6:**
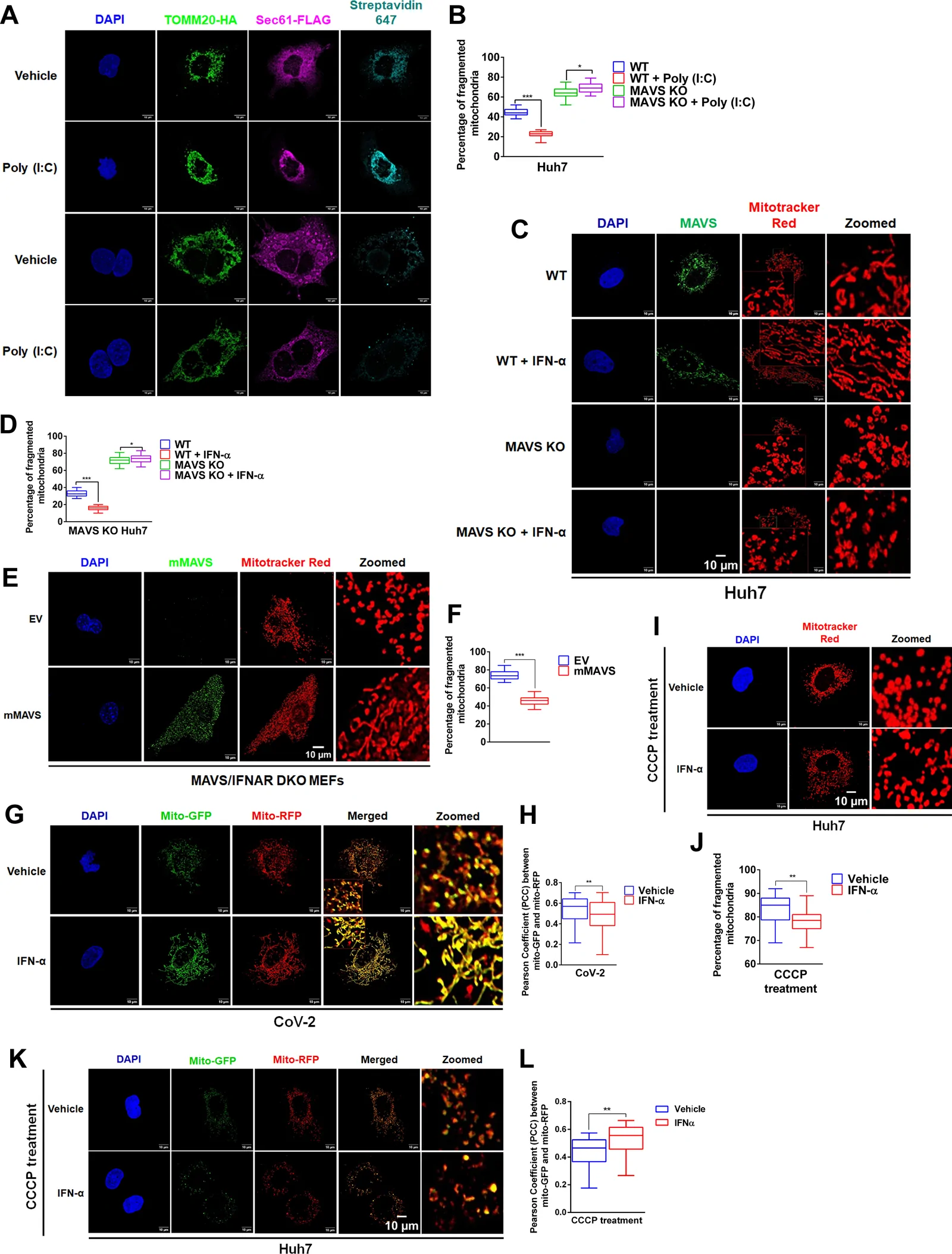
MAVS maintains mitochondrial integrity independently of IFN signaling. (**A**) Super-resolution images showing HA-tagged TOM20 (mitochondria), FLAG-tagged Sec61 (ER), and Streptavidin-647 signal (labeling biotinylated MAM contact sites generated by the Contact-ID proximity biotinylating system) in vehicle or poly(I:C)-treated WT (top two rows) and MAVS KO (bottom two rows) cells. (**B**) Quantification of mitochondrial fragmentation from the experimental setup described in panel **A**. (**C**) Confocal images analyzing mitochondrial fragmentation in IFN-α-treated Huh7 WT and MAVS KO cells and (**D**) corresponding quantification. (**E**) Confocal images analyzing mitochondrial fragmentation in MAVS/IFNAR DKO MEFs overexpressing mMAVS and (**F**) corresponding quantification. (**G**) Confocal images analyzing mitophagy in CoV-2-infected, IFN-α-treated Huh7 cells and (**H**) corresponding quantification. (**I**) Confocal images studying mitochondrial fragmentation in CCCP- and IFN-α-treated Huh7 cells and (**J**) quantification of mitochondrial fragmentation from panel **I**. (**K**) Confocal images showing mitophagy in CCCP- and IFN-α-treated Huh7 cells and (**L**) quantification of mitophagy from images in panel **K**. Scale bars, 10 µm, and *n* = 50 cells unless otherwise indicated. Data are presented as mean ± SEM, with *, **, and *** denoting *P* < 0.05, 0.01, and <0.001, respectively.

Finally, to verify whether the MAVS-dependent intrinsic antiviral activity involves mitochondrial remodeling independent of canonical signaling outputs, we monitored mitochondrial responses to RNA and interferon stimulation. Poly(I:C) induced pronounced mitochondrial elongation in WT cells, whereas MAVS-KO cells failed to remodel mitochondrial networks in response to RNA sensing (Fig. 6A and B). Similarly, IFN-α treatment elongated mitochondria in WT Huh7 cells but had no effect in the absence of MAVS (Fig. 6C and D). Given that mitochondrial elongation has been linked to enhanced mitochondrion–ER communication (27, 55), we next assessed mitochondrion–ER contact sites (MAMs) using Contact-ID, a split-BioID reporter system based on a TOM20–Sec61 pair that labels mitochondria in the context of mitochondrion–ER junctions (56). Considering that MAMs function as upstream platforms that drive MAVS-dependent IFN production (27, 55), and since IFN signaling failed to elongate mitochondria in MAVS-KO cells (Fig. 6C and D), this assay was specifically designed to probe the upstream poly(I:C)/MAVS axis. In line with our mitochondrial elongation results, poly(I:C) treatment in WT cells induced a strong increase in MAMs but failed to do so in MAVS KO cells (Fig. S6A through C). Together, these data indicate that MAVS is critical for both RNA- and IFN-triggered mitochondrial remodeling.

We further validated this IFN-independence in MAVS/IFNAR1 double-knockout cells. MAVS supplementation restored mitochondrial network organization in this IFN-deficient background (Fig. 6E and F). Furthermore, IFN-α treatment during SARS-CoV-2 infection failed to restore either mitochondrial membrane potential (Fig. S6D and E) or reduce mitophagosome accumulation (Fig. 6G and H) to the extent observed with MAVS supplementation (Fig. 4P through S). To determine whether interferon can broadly counteract mitochondrial damage independent of viral infection, we examined mitochondrial morphology following CCCP-induced depolarization. IFN-α treatment failed to prevent CCCP-induced mitochondrial fragmentation (Fig. 6I and J), membrane depolarization (Fig. S6F and G), and mitophagosome accumulation (Fig. 6K and L), indicating that interferon signaling is insufficient to suppress mitochondrial damage even in a non-viral context. These results confirm that the mitochondrial stabilization by MAVS is completely independent of IFN-signaling activities. Collectively, these findings establish MAVS as a regulator of intrinsic antiviral immunity that preserves mitochondrial integrity and import competence, thereby restricting viral replication independently of canonical interferon and NF-κB signaling pathways.

## DISCUSSION

**Fig 7:**
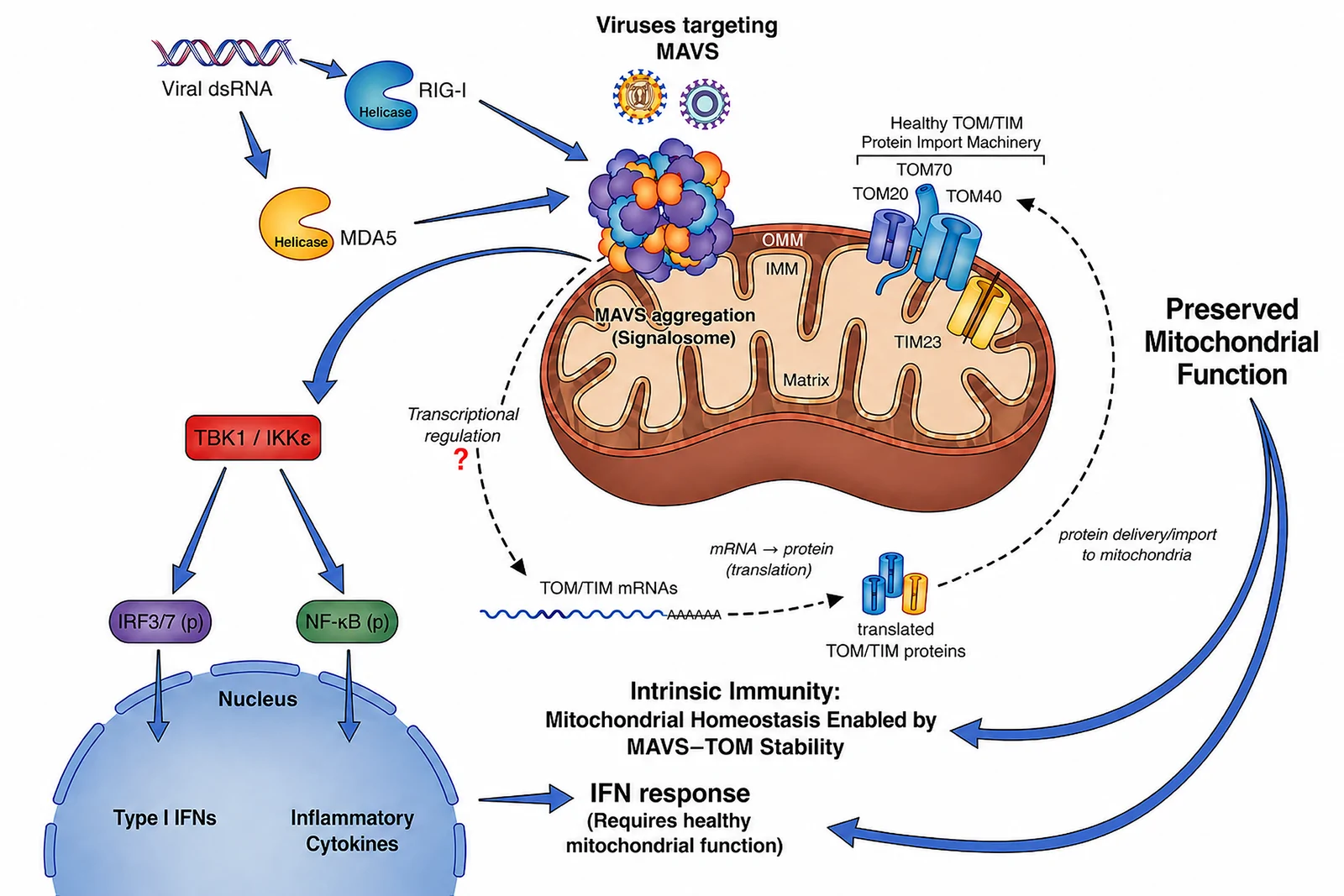
MAVS-dependent antiviral signaling is coupled to mitochondrial protein import and organelle homeostasis. Viral RNA sensing by RIG-I and MDA5 activates MAVS on the OMM, driving oligomerization into higher-order signalosomes that trigger TBK1/IKKε → IRF3/7 → type I IFN and NF-κB → inflammatory cytokine outputs. In parallel, we identify a potent IFN-independent antiviral mechanism: MAVS transcriptionally regulates TOM/TIM complex components to maintain mitochondrial protein import competence and suppress PINK1-dependent mitophagy. MAVS loss depletes TOM/TIM subunits, impairs import, elevates mitophagy, and compromises both antiviral arms. RNA viruses exploit this by disrupting MAVS aggregation and mitochondrial localization, simultaneously blunting IFN signaling and destabilizing mitochondrial homeostasis. The dashed arrow denotes the transcriptional link between MAVS and TOM/TIM mRNAs; the upstream mechanism remains an open question.

Antiviral defense is layered: in addition to inducible innate immune signaling that culminates in interferons and cytokines, cells deploy intrinsic, cell-autonomous defenses that restrict infection by maintaining a non-permissive intracellular state. Intrinsic immunity is characterized by specialized host restriction factors that employ discrete mechanisms to target specific stages of the viral life cycle. Two unifying themes emerge from our study. First, mitochondrial fitness—encompassing import competence, membrane potential, and bioenergetic integrity—is a prerequisite for antiviral restriction, not merely a passive housekeeping function. Second, MAVS serves as the molecular bridge linking organelle health to broad-spectrum antiviral defense, operating through both IFN-dependent RLR signaling and IFN-independent cell-intrinsic mechanisms. Here, we show that mitochondria play crucial roles in enacting a potent intrinsic immunity independent of IFN. MAVS, previously known for its role in coordinating IFN production, is a decisive player in intrinsic immunity. We show that MAVS orchestrates this by safeguarding mitochondrial integrity by ensuring proper mitochondrial protein import, thereby restraining PINK1-driven mitophagy. This IFN-independent mitochondrial safeguard, exploited by RNA viruses (like SARS-CoV-2 or JEV), enforces viral restriction even when canonical IFN outputs are weak or blocked. Given that the RLR pathway is also critically dependent on mitochondrial integrity for signalosome complex assembly, our findings suggest that MAVS has a mitochondrial maintenance function that operates in parallel to, and is required for, effective antiviral immunity. Whether this function is ancestral to, or co-evolved with, the signaling role of MAVS remains an open question (Fig. 7).

MAVS possesses an extensive interactome map that shows its potential interactions with over 250 proteins (57). Of these, interactions with TOM20 and MFN1/2 indicated a potential influence by this molecule on mitochondrial homeostasis. Indeed, our deep sequencing indicated strongly in that direction. We demonstrate that MAVS is critical for proper assembly of the TOM/TIM complex. Mechanistically, our data support a model in which MAVS preserves the integrity of the mitochondrial import apparatus and its higher-order organization on the outer membrane. Loss of MAVS selectively depletes key TOM/TIM receptor and channel components, perturbs TOM/TIM assemblies, and compromises import, which are defects that are expected to destabilize respiratory function and membrane potential. Import failure provides a direct route to PINK1 stabilization and engagement of mitophagy, linking MAVS-dependent mitochondrial competence to suppression of PINK1-dependent quality control programs that otherwise remodel (and potentially deplete) the mitochondrial network.

Many RNA viruses antagonize MAVS to suppress antiviral signaling; our findings indicate that this interference carries a second, underappreciated consequence, which is destabilization of mitochondrial homeostasis itself. During SARS-CoV-2 infection, MAVS fails to undergo productive aggregation, coinciding with mitochondrial fragmentation, loss of membrane potential, PINK1 stabilization, and elevated mitophagosome formation. Notably, SARS-CoV-2 ORF9b directly targets TOM70 to disrupt HSP90–TOM70 interaction and suppress MAVS-dependent signaling (26, 30), suggesting that viral subversion of the TOM import apparatus may simultaneously disable IFN induction and exacerbate the mitochondrial import defects. Our results indicate that these changes are not unique to SARS-CoV-2 infection, but represent many such viruses that promote mitophagy, as we showed in JEV. These defects closely phenocopy MAVS deficiency, suggesting that viral disruption of MAVS assembly simultaneously dampens canonical antiviral signaling and drives mitochondria into an import-compromised, quality-control-engaged state. Together, this provides a tight mechanistic link between viral immune evasion at the level of MAVS and infection-associated mitochondrial dysfunction.

A key implication of our findings is that mitophagy should not be viewed solely as a source of membrane platforms for viral replication. Instead, loss of mitochondrial mass and integrity can itself erode antiviral competence, particularly in non-immune cells where mitochondrial fitness underpins both signaling capacity and metabolic flexibility. Consistent with this view, experimentally inducing mitophagy enhanced replication of viruses that benefit from mitochondrial pruning, such as SARS-CoV-2 and JEV, whereas viruses that rely on intact mitochondrial function or employ distinct remodeling strategies, exemplified by DENV, did not benefit and were instead impaired. These observations underscore that the consequences of mitophagy are virus-specific and tightly coupled to how individual pathogens exploit or tolerate mitochondrial dysfunction.

Conceptually, our study illustrates that mitochondrial homeostasis is an upstream determinant of both IFN-dependent and IFN-independent antiviral responses, with MAVS acting at the interface of these two layers. The mitochondrial regulatory landscape—encompassing both structural maintenance and metabolic homeostasis—is intrinsically coupled to the cell’s capacity for broad-spectrum antiviral defense. Specifically, we highlight how fundamental processes, such as mitochondrial protein import, function as critical determinants of the cellular antiviral state. Notably, MAVS-dependent changes in TOM/TIM transcript abundance suggested that MAVS is associated with far-reaching control over mitochondrial architecture. Whether MAVS directly regulates transcription of TOM/TIM components or whether this reflects indirect consequences of altered mitochondrial homeostasis remains to be determined; however, the convergent downregulation across two independent genetic models—human KO cells and murine KO MEFs—supports a consistent, MAVS-dependent regulation of import machinery gene expression. Given the extensive inter-organellar communication networks, these MAVS-dependent mitochondrial alterations likely reverberate throughout the cell, impacting the functional homeostasis of associated organelles. The evolutionary pressure on viruses to develop sophisticated strategies for subverting MAVS further underscores its pivotal role in maintaining the host environment. Ultimately, by unraveling these multifaceted regulatory nodes, our study significantly advances the conceptual framework of the innate antiviral response.

“Intrinsic immunity” has traditionally been defined as constitutive, cell-autonomous restriction mediated by pre-existing antiviral factors. Our findings extend this concept by identifying organelle fitness as a major determinant of intrinsic antiviral competence, with MAVS acting as a regulator of mitochondrial integrity at the organelle surface. This role may be particularly consequential in non-immune cell types such as epithelial cells and fibroblasts, where preservation of mitochondrial networks and mitochondria–ER contact sites can determine the baseline capacity for antiviral signaling and metabolic adaptation, even before cytokine amplification occurs. An additional implication of our data is that mitochondrial safeguarding by MAVS may indirectly modulate inflammatory outcomes. Severe mitochondrial perturbation can promote danger signaling, including mtDNA release and inflammasome activation. However, the primary phenotypes we observe—import failure, loss of membrane potential, PINK1 stabilization, and engagement of mitophagy—arise upstream of overt inflammatory cell death. We therefore favor a model in which MAVS-dependent mitochondrial integrity restrains excessive quality control activation and secondary danger signaling by preventing escalation of mitochondrial damage, a function that may be especially relevant in epithelial immune circuits during viral infection.

MAVS-KO is widely used in the field as a proxy for loss of IFN signaling during infection; however, our findings indicate this interpretation warrants caution. MAVS-KO simultaneously ablates both IFN-dependent antiviral signaling and the IFN-independent mitochondrial maintenance function described here, such that phenotypes attributed to loss of IFN signaling in MAVS-KO systems may, in some cases, instead reflect loss of mitochondrial homeostasis. In line with this, a complementary control—a MAVS variant that retains CARD function but cannot respond to the upstream RLRs would help separate these activities (oligomerization of MAVS with upstream RIG-I/MDA-5 signaling) and strengthen our understanding of CARD domain dependence, but no such selective variant is currently characterized (8, 58).

Three limitations of this study merit consideration. First, MAVS supplementation likely “preconditions” cells, and the extent to which MAVS can be therapeutically restored after infection onset may be constrained by virus-induced stress or cytotoxicity. Second, the mechanism by which MAVS preserves mitochondrial import machinery remains unclear and may involve TOM/TIM scaffolding, membrane organization, or maintenance of mitochondrial integrity. Restoring TOM/TIM function in MAVS-knockout cells could test whether import competence alone rescues antiviral activity. However, strict complex stoichiometry and interdependence among TOM, TIM22, and TIM23 make single-subunit overexpression unlikely to restore functional import (59–61). Coordinated reconstitution therefore remains an important future direction. Our analysis of MAVS aggregation topology is also limited by the lack of a MAVS-independent outer mitochondrial membrane marker, as TOM20 and TOM70 are themselves regulated by MAVS. This prevented us from determining whether MAVS interactions occur within or between mitochondria. However, recent structural work indicates that both configurations occur, with intra-mitochondrial fibrils forming the dominant signaling architecture (62). Third, while our data demonstrate a consistent association between MAVS-dependent TOM complex regulation, import competence, and antiviral restriction, the precise causal relationship between these events, specifically whether import competence itself is the antiviral effector, or whether MAVS exerts additional mitochondrial functions independent of the TOM complex remains to be established. Future experiments using PINK1 inhibition or mitophagy blockade in MAVS-deficient cells will be necessary to dissect this chain.

Together, our findings reveal an additional layer of antiviral function of MAVS that operates alongside its established signaling role and suggest that viral disruption of MAVS assembly has consequences for mitochondrial homeostasis beyond the suppression of IFN induction. This organelle-centric function of MAVS provides a mechanistic bridge between viral immune evasion and mitochondrial remodeling and suggests that strategies aimed at preserving mitochondrial fitness may offer therapeutic leverage even when canonical interferon pathways are compromised.

## MATERIALS AND METHODS

### Antibodies

MDA-5 (Cat #5321), RIG-I (Cat #3743), human MAVS (Cat #24930), rodent MAVS (Cat #4983), phospho-IRF-3 (Cat #4947), IRF3 (Cat #11904), phospho-STAT1 (Cat #14994), STAT1 (Cat #9167), IFIT1 (Cat #14769), phospho P65 (Cat #3033), P65 (Cat #8242), HA-tag (Cat #3724 and #2367), FLAG-tag (Cat #14793), LC3 A/B (Cat #12741), TOM70 (Cat #65619), TOM20 (Cat #42406), TOM22 (Cat #90704), and TOM40 (Cat #55959) were purchased from Cell Signaling Technologies (CST). TIM23 (Cat #sc-514463) and TOM40 (Cat #sc-365467) were from Santa Cruz Biotechnology. JEV NS1 (Cat #GT1410) and DENV NS1 (Cat #GTX124280) antibodies were from Genetex. dsRNA J2 antibody (Cat #10010200) was procured from Scicons. Phospho-PINK1 (Cat #PA5-105356), PINK1 (Cat #PA5-86941), CoV-2 nucleocapsid (Cat #MA5-36272), GAPDH (Cat #MA5-15738), β-tubulin (Cat #MA5-16308), ACE2 (Cat #MA5-32307), and Alexa Fluor 647 conjugated with streptavidin (CAT #S21374) were procured from Thermo Fisher Scientific. Goat anti-rabbit HRP-conjugated (Cat #115-035-146), Alexa Fluor 488 conjugated (Cat #115-545-146), Alexa Fluor 647 conjugated (Cat #115-605-146), and goat anti-mouse HRP conjugated (Cat #115-035-003), Alexa Fluor 488 conjugated (Cat #115-545-003), Alexa Fluor 594 conjugated (Cat #111-585-006), and Alexa Fluor 647 conjugated (Cat #115-605-003) were purchased from Jackson ImmunoResearch.

### Inhibitors and reagents

Human IFNα 2a (Cat #11100) was purchased from PBL Assay Science. Poly (I:C) (Cat #P1530), BAY-11-7082 (NF-κB inhibitor, Cat #196870), baricitinib (JAK-STAT inhibitor, Cat #SBR00076), and carbonyl cyanide m-chlorophenylhydrazone (Cat #C2759) were from Sigma-Aldrich. Human type I IFN neutralizing antibody mixture (Cat #39000) was from PBL Assay Science. Human IFN-lambda was from R&D Systems (Cat # 1598-IL), Human IFN alpha ELISA Kit (Cat #BMS216), and JC-1 dye (Cat #T3168) were from Invitrogen. MitoTracker Red (CMX-ROS) dye (Cat #9082) was from Cell Signaling Technologies.

### Plasmids and cloning

Mammalian expression vector, pcDNA4/TO, was from Invitrogen. Mammalian expression vector pUNO and HA-MAVS were from Invivogen. HA-ΔCARD-MAVS was cloned as reported previously (44). Mouse MAVS was amplified using cDNA from MEFs and cloned into pcDNA4/TO. Mitophagic flux was assessed using a p-mito-mRFP-EGFP plasmid (Plasmid pAT016) (63). Mitochondrial protein import assay was performed using an MTS-GFP fusion protein cloned into the pcDNA/4TO plasmid (64). MAM formation was assessed using Contact-ID—a split-BioID system (56).

### Generation of MAVS and IFNAR1 double KO MEFs

All mice were bred and maintained under specific pathogen-free conditions at the CCMB Animal Facility. Double knockout mice for MAVS and IFNAR1 were generated using MAVS KO males (B6129SF2/J, #008634, Jackson Laboratory) and IFNAR1 KO females (C57BL/6J, #028288). Appropriate animal ethical clearances were obtained from the Institutional Animal Ethics Committee of CCMB, and their guidelines were strictly followed. First-generation (G1) heterozygous offspring (MAVS^+/−^; IFNAR1^+/−^) were genotyped and interbred to produce G2 mice, including double homozygotes (MAVS^−/−^; IFNAR1^−/−^). Genomic DNA was prepared from their tail samples (1–2 mm), which were genotyped by PCR using gene-specific primers targeting wild-type and mutant alleles. Amplicons were analyzed via agarose gel electrophoresis. MAVS KO yielded a 350 bp band, IFNAR1 KO a ~450 bp band, and heterozygotes showed both bands. Thermal cycling conditions were optimized separately for each gene.

### Cell culture

Caco-2 cells were obtained from ATCC, while Huh7 cells were generously provided by Ralf Bartenschlager (65). A549 WT and MAVS KO cells were from Invivogen. MEFs were isolated from WT, MAVS KO, and MAVS:IFNAR1 DKO mice following the established protocol (66). All cells, except for Caco-2 (20% FBS), were maintained in DMEM (Gibco) supplemented with 10% FBS (Gibco), 1× penicillin–streptomycin cocktail (Gibco), and 1× non-essential amino acids (Gibco) at 37°C with 5% CO2. Cells were routinely passaged at 70%–80% confluency, and mycoplasma contamination was periodically monitored.

### Generation of MAVS KO Huh7

MAVS KO Huh7 cells were generated using the CRISPR-Cas9 system. Single-guide RNAs (sgRNAs) targeting exon 6 of the MAVS gene were inserted into the PX459 vector containing WT Cas9 (Addgene, Cat #62988). Huh7 cells were transfected with the sgRNA-containing PX459 plasmids using Lipofectamine 3000. Following transfection, puromycin selection was used to enrich transfected cells. Knockout validation was performed by immunoblotting in multiple clones to confirm the loss of MAVS protein. Successfully edited clones were expanded and verified as MAVS knockout Huh7 cells. sgRNA primers used are given in Table 1.

**TABLE 1:** sgRNA primers

Exon details	sgRNA targeting exon 6 of MAVS
Target sequence	CGATGCCCGCTGGAGGTCAGAGGGCAAA
Complementary sequence	CACCGCTACGGGCGACCTCCAGTCTCCC

### JEV, DENV, and SARS-CoV-2 isolates

The five variant isolates of SARS-CoV-2 used in this study were isolated at the Centre for Cellular and Molecular Biology in the biosafety level 3 facility (10). The viruses were propagated in Vero (CCL-81) cells grown in serum-free DMEM (SFD). JEV and DENV were propagated as previously described (44).

### Virus infection and quantification

Cells were infected at a multiplicity of infection of 1 for 3 h in serum-free conditions after which the media was replaced with complete media and further incubated until the time of harvesting as per experimental requirement. During harvesting, the cells were first trypsinized and collected separately for protein and RNA studies. Titration for SARS-CoV-2, JEV, and DENV was done as described (10, 44). For overexpression studies, cells were seeded to reach 50%–60% confluency. Transfection mix containing Opti-MEM, Lipofectamine 3000, along with plasmid of interest was prepared according to the manufacturer’s protocol, added to cells, and incubated for 6 h. Later, the transfection mix was replaced with cDMEM and further incubated for 18 h. After 24 h of transfection, cells were infected with virus for 3 h and further incubated in fresh cDMEM until they were harvested for analyses. A similar approach was used for Poly(I:C) transfections.

For IFN-α and IFN-λ1 treatment, cells were serum-starved at 80%–85% confluency in SFD for 3 h. Later, SFD was replaced with fresh SFD containing 1,000 U/mL IFN-α or IFN-λ1 for 1 h. Following this, the cells were infected for 3 h and incubated further with fresh cDMEM containing either phosphate-buffered saline (PBS; vehicle) or 1,000 U/mL of IFN-α or IFN-λ1 for the subsequent 21 h. Cells and culture supernatants were harvested at 24 hpi and used for further analysis.

For baricitinib and BAY-11-7082 treatments, cells were treated with the inhibitors at 3 hpi and incubated for the subsequent 21 h. For CCCP treatments, the cell culture medium was removed, and cells were supplemented with CCCP-containing cDMEM 2 h and 6 h before harvesting, respectively. Cells and cell culture supernatants were harvested and used for further analysis.

### Real-time quantitative RT-PCR

Cellular RNA samples were prepared using an MN NucleoSpin RNA kit (TaKaRa, Cat #740902). Equal quantities of RNA were reverse-transcribed using Primescript 1st-strand cDNA synthesis kit (TaKaRa, Cat #6110) following the manufacturer’s protocol. Fifty nanograms of cDNA was used for quantification using TB Green Premix (TaKaRa, Cat #RR820) on a LightCycler 480 instrument (Roche). Transcripts of the host origin were normalized against GAPDH. qRT-PCR to quantify SARS-CoV-2 RNA was performed on a Roche LightCycler 480 using an nCOV-19 reverse transcription-PCR (RT-PCR) detection kit from Q-Line Molecular (Cat #COVIDM96PS). Relative fold changes between the experimental and control samples (2^−ΔΔCT^) were calculated and represented in the graphs. Primer sequences for *IFNB1* and *DENV* are reported earlier (44). The following primers were used for qRT-PCR of *IFNL1*: forward primer, GGTGACTTTGGTGCTAGGCT; reverse primer, TGAGTGACTCTTCCAAGGCG.

### Immunoblotting

Cell pellets were lysed in 1× Nonidet P-40 lysis buffer (1% Nonidet P-40, 50 mM Tris-HCl, 150 mM NaCl [pH 7.5], EGTA, 1 mM sodium orthovanadate, 10 mM sodium pyrophosphate, 100 mM NaF, and 1 mM PMSF), incubated on ice for 20 min with intermittent vortexing, and centrifuged at 13,000 rpm for 15 min at 4°C. The supernatants containing the proteins were collected and quantified using BCA reagents (Pierce, Cat #23225). Lysates were mixed with 6× denaturing dye, and the proteins were resolved using SDS-PAGE and transferred to PVDF membranes. The membranes were blocked in 5% BSA dissolved in 1× TBST before the addition of primary antibodies. Primary antibodies against the proteins of interest were diluted in the blocking buffer, added to the membrane, and incubated overnight at 4°C. Later, the membranes were washed in 1× TBST, secondary antibodies conjugated with HRP were added, and the blots were developed on a Bio-Rad ChemiDoc MP system using Clarity Western ECL substrate (Bio-Rad, Cat #1705061) and Clarity Max Western ECL substrate (Bio-Rad, Cat #1705062) chemiluminescent substrate kits. Relative fold changes in protein expression between the experimental and control samples were determined and depicted in the graphs.

### Blue native-PAGE

To assess TOM complex stability and abundance, Blue native PAGE (BN-PAGE) was performed following established protocols (67). Briefly, protein content in mitochondrion-enriched fractions was quantified using Bradford Assay reagent, then samples were solubilized as described previously: digitonin (Sigma-Aldrich; Cat #D141-500MG) was added at 12 g per g of mitochondrion-enriched fraction and incubated for 10 min on ice, followed by 30-min centrifugation at 13,000 × *g* to collect the supernatant. The protein concentration of this supernatant was re-measured by Bradford, then 5% G-250 sample dye was added at 6 g per g of mitochondrial-soluble fraction, and glycerol was adjusted to a final 10%.

Sample (20–30 µg) was loaded onto 4%–15% Mini-PROTEAN TGX Precast Protein Gels (Bio-Rad; Cat#4561086) and run with anode buffer (50 mM Bis-Tris HCl, pH 7.0) and blue cathode buffer (50 mM Tricine, 15 mM Bis-Tris HCl, pH 7.0, 0.02% Coomassie Brilliant Blue G-250). Electrophoresis was performed at 100 V until entry into the separating gel, then 150 V until the dye front reached ~1/3 of the gel, after which the blue cathode buffer was replaced with dye-free cathode buffer and the run continued at 250 V until the dye front reached the gel end.

### Seahorse Mito Stress Assay

Mitochondrial oxygen consumption was assessed using a Seahorse XFe 24 Analyzer. In summary, 50,000 cells per well were plated in XFe 24 plates, washed twice with DPBS, and incubated for 1 h at 37°C in a CO₂-free incubator with XFe DMEM assay medium containing 10 mM glucose, 2 mM L-glutamine, and 1 mM sodium pyruvate. Oligomycin (3.5 μM), FCCP (3 μM), and a combination of antimycin A (1.5 μM) with rotenone (1.5 μM) were introduced through reagent ports A, B, and C, respectively. Measurements were taken for basal OCR, ATP-linked OCR, and proton leak, with all values normalized to total protein content determined using the Bradford assay.

For overexpression studies, cells were transfected with the appropriate plasmids in 6-well plates for 24 h, followed by trypsinization and reseeding at a density of 50,000 cells per well in XFe 24-well plates. After reseeding, the same Seahorse assay procedure was carried out as previously described.

### Preparation and NMR analysis of metabolites from cell pellets

Cells incubated with medium containing [1,6-^13^C] glucose for 45 min were harvested and stored at −80°C for further analysis. Metabolites were then extracted from harvested cells using an ethanol-based extraction protocol (68). In brief, the cell pellet was homogenized in methanol–HCl (0.1 N) (2:1 vol/wt) using a battery-operated tissue homogenizer. [2-^13^C]glycine (50 μL, 2 mmol/L) was added as an internal reference, followed by homogenization with ethanol (90%)–phosphate buffer (6:1, vol/vol). The homogenate was centrifuged (20,000 × *g*, 45 min, 4°C), and the supernatant was pH-adjusted and freeze-dried. The lyophilized powder was dissolved in phosphate buffer (25 mmol/L, pH 7.0, 80% D_2_O) containing sodium trimethylsilyl propionate (0.25 mmol/L) for NMR measurements. Total and ^13^C-labeled concentrations of metabolites were measured in a ^1^H-[^13^C]-NMR spectrum on a 600 MHz NMR spectrometer (69). The ^13^C-edited spectrum was obtained through spin-echo acquisition with and without a ^13^C inversion pulse. Metabolite levels were quantified relative to [2-^13^C] glycine, and ^13^C enrichment was corrected for natural abundance (subtracting 1.1%). The rate of glucose consumption (MR Glc) is calculated from the ^13^C labeled trapped into different metabolites (70). The rate of ATP synthesis was calculated as described previously using an approximation of 2.5 and 1.5 ATP yield for each molecule of NADH and FADH, respectively: ATP syn = (2 NADH + 2 ATP) × Lac Syn + (10 NADH + 2 FADH_2_ + 4 ATP) × MR Glc (Lac Syn is the rate of lactate synthesis).

### Microscopy experiments

#### JC-1 staining

In total, 50,000 cells were seeded in 6-well plates and allowed to adhere overnight. Cells were subjected to experimental conditions (virus infection, treatments, overexpression, or phenotype comparison), washed twice with SFD, and stained with 10 µM JC-1 dye in SFD for 20–30 min at 37°C in the dark. After staining, cells were washed twice with SFD, replenished with fresh SFD, and imaged using a Zeiss Axio Observer 3 microscope with FITC and mCherry filters to visualize green JC-1 monomers and red aggregates. ΔΨm was expressed as the JC-1 ratio = (Red_mean − Red_bg)/(Green_mean − Green_bg). Ratios were calculated per cell and summarized as mean ± SEM across biological replicates.

#### Mitotracker red staining

In total, 50,000 cells were seeded on coverslips in 6-well plates, subjected to experimental conditions, washed twice with SFD, and incubated with 100 nM Mitotracker Red or Mitotracker Green in SFD for 20–30 min at 37°C in the dark. After staining, cells were washed twice with SFD, fixed with 4% formaldehyde for 30 min, washed with PBS, and stored at 4°C before immunostaining.

#### Mito-GFP-RFP reporter assay

In total, 50,000 cells were seeded on coverslips, transfected with 2 µg of Mito-GFP-RFP reporter plasmid, and subjected to experimental conditions 24 h post-transfection. Cells were washed twice with PBS, fixed with 4% formaldehyde for 30 min, washed with PBS, and stored at 4°C before immunostaining. Mitophagy was quantified as the PCC between the GFP and RFP channels, with lower PCC values indicating increased mitophagy due to loss of GFP fluorescence in acidic lysosomal compartments, while the RFP signal is retained. PCC values were calculated per cell and averaged across cells and independent biological replicates.

#### Mitochondrial protein import assay

In total, 50,000 cells were seeded on coverslips, transfected with 2 µg of MTS-GFP reporter plasmid and mCherry as a transfection normalization control. After 12 h of transfection, cells were washed twice with PBS, fixed with 4% formaldehyde for 30 min, washed with PBS, and immunostained. For WT + CCCP conditions, cells were transfected with MTS-GFP for 10 h, followed by CCCP treatment for 2 h before fixing the cells. For MAVS supplementation assays, empty vector, FL-MAVS, and ∆CARD MAVS were co-transfected along with MTS-GFP and mCherry and were fixed 12 h post-transfection before proceeding to immunostaining.

#### MAM formation assay

In total, 50,000 cells were seeded on coverslips, co-transfected with 2 µg of HA-TOM20-BirA and FLAG-Sec61-BirA reporter plasmid pair (Contact ID). After 24 h of transfection, cells were biotinylated with 50 μM of biotin and subjected to experimental conditions 24 h post-biotin treatment. Cells were washed twice with PBS, fixed with 4% formaldehyde for 30 min, washed with PBS, and stored at 4°C before immunostaining.

#### Immunostaining

Fixed coverslips were washed twice with PBS, permeabilized (0.05% Tween-20, 0.25% Triton X-100 in PBS) for 10 min, and washed three times with PBST. Blocking was performed with 1% BSA and glycine in PBST. Coverslips were incubated overnight at 4°C with primary antibodies diluted in 1% BSA in PBST. After washing, fluorophore-conjugated secondary antibodies were added, followed by three washes with PBST. Coverslips were mounted with DAPI-containing anti-fade mountant (Cat #H-1200-10) and analyzed by microscopy.

#### Imaging and analysis

Confocal microscopy was performed using an Olympus FV3000 and Leica SP8 microscopes (60× oil immersion objective for Olympus FV3000, 63× oil immersion objective for Leica SP8, 2.5× optical zoom). Super-resolution imaging for the contact-ID assay was conducted using a ZEISS Elyra 7 system with Lattice SIM² (63× objective, variable zoom). Mitochondrial fragmentation and volume analysis of clumped mitochondria (in SARS-CoV-2 and JEV-infected cells) were analyzed using Imaris software. JC-1 red/green ratios and co-localization (e.g., GFP-RFP, MAVS-mitochondria) were quantified with ImageJ. Binary HA-TOM20-FLAG-Sec61 intersections for MAMs were also calculated using ImageJ.

#### Intersection density and spatial proximity analysis

Confocal images of Huh7 WT and MAVS KO cells were acquired using the Olympus FV3000 system. Post-acquisition, image stacks containing TOM20, TOM70, and Mitotracker Red channels were deconvolved using the DeconvolutionLab2 plugin in ImageJ, applying the Richardson–Lucy algorithm (20 iterations). Following deconvolution, TOM20 and TOM70 channels were merged, thresholded, and intersection masks were generated using ImageJ’s Image Calculator function. Intersections were quantified using the Analyze Particles tool. Mitochondrial area was determined from the MitoTracker Red channel, and intersection density was calculated as the number of TOM20–TOM70 intersections per unit mitochondrial area. For spatial proximity analysis, deconvolved TOM20 and TOM70 images were thresholded, and centroid-to-centroid distances were computed using the MorphoLibJ plugin in ImageJ.

#### Cyto/Mito GFP accumulation analysis

Confocal image stacks were acquired with an Olympus FV3000 system equipped with a 60× oil immersion objective, using sequential excitation to record DAPI, MTS-GFP, mCherry, HA (when present), and TOM20 channels. Raw files were imported into Fiji (Bio-Formats). Binary masks were generated by automated thresholding: the mCherry channel yielded the whole-cell mask, TOM20 the mitochondrial mask, and DAPI the nuclear mask. A cytosolic mask was produced by subtracting the nuclear and mitochondrial masks from the whole-cell mask (Image Calculator, “Subtract create”). Mean GFP fluorescence was then measured within (i) the cytosolic mask and (ii) the TOM20-defined mitochondrial mask. Import efficiency was expressed as the Cyto/Mito ratio: Cyto/Mito ratio = mean GFP cytosol/mean GFP mitochondria.

All threshold levels, arithmetic operations, and measurement parameters were applied identically to every image and condition.

### Next-generation sequencing

Library preparation was done using the MGIEasy RNA library prep set (MGI) according to the manufacturer’s instructions. In brief, 500 ng total RNA was used as starting material from which rRNA was depleted using a Ribo-Zero Plus rRNA depletion kit (Illumina). The rRNA-depleted samples were fragmented and reverse-transcribed, and the second strands were synthesized. DNA was then purified using DNA clean beads provided in the kit, followed by end repair and A-tailing. Barcoding and adaptor ligation were performed, and the samples were purified. Samples were amplified using adaptor-specific primers and quantified using a Qubit dsDNA high-sensitivity kit (Thermo Scientific). Sample fragment size was determined using a 4200 Tape Station (Agilent). The samples were denatured, and single-stranded circular DNA strands were generated. Furthermore, rolling circle amplification was performed to generate DNA nanoballs. The samples were subsequently loaded onto the flow cells, and paired-end RNA sequencing was performed on the MGI 2000 (MGI) platform for a read length of 100 bases.

### Data processing and analysis

Raw sequencing reads were processed using Cutadapt (71) to remove MGI adapters and low-quality sequences. Reads with a quality score below 20 and a length shorter than 36 bp were discarded. The remaining high-quality reads were mapped to the human reference genome (GRCh38) using HISAT2 with default parameters (72). Uniquely aligned reads were quantified using the featureCounts function from the Subread package (73), based on a GTF annotation file downloaded from Ensembl (74), which contained read count data for 60,683 genes. Genes with fewer than 10 total read counts across all samples were excluded from further analysis. Differential gene expression analysis was performed using DESeq2 (75), with genes considered differentially expressed if they had a *P*-value <0.05 and |log2FC| ≥0.58. For cross-species comparison, MAVS-OE Huh7 cells and MAVS-KO MEFs were each analyzed independently against their own species-matched controls in separate DESeq2 runs; human and mouse DEG sets were subsequently aligned by orthologous gene identity using homolog mapping for joint visualization. For mitochondrial import RNA-seq data sets, mitochondrial DEGs were defined as protein-coding genes (Ensembl annotation) that reached statistical significance by DESeq2, cross-referenced against the MitoCarta 3.0 database to identify mitochondrially associated genes. Both nuclear-encoded and mitochondrially encoded protein-coding genes were included; tRNA and rRNA biotypes were excluded.

### Functional enrichment analysis

Functional enrichment analysis was conducted using clusterProfiler (76) to identify enriched GO terms. To refine the results, similar enriched terms were merged using the simplify function of ClusterProfiler, with a similarity cut-off of 0.5. The *P*-value was utilized as a criterion for selecting representative terms, while the min method was applied for feature selection. The Wang method was employed to measure similarity. Finally, the top five enriched GO terms for the genes were visualized.

### Statistical analysis

For each experiment, at least three independent replicates (until specified) were used to calculate mean ± SEM and plotted graphically wherever indicated. Statistical significance was measured using a two-tailed, unpaired Student *t*-test, and the resultant *P* values were represented as *, **, and *** indicating *P* values ≤0.05, 0.01, and 0.001, respectively.

### Biosafety statement

All infection protocols in this study were approved by the Institute Biosafety Committee of CSIR-CCMB.

## Data Availability

All data pertaining to this article are included within the article. The RNA sequencing (RNA-seq) data have been submitted to the Gene Expression Omnibus (GEO) database and are available under the accession number GSE293066.

## SUPPLEMENTAL MATERIAL

Supplemental figures mbio.02071-26-s0001.docx Fig. S1 to S6.

ASM does not own the copyrights to Supplemental Material that may be linked to, or accessed through, an article. The authors have granted ASM a non-exclusive, world-wide license to publish the Supplemental Material files. Please contact the corresponding author directly for reuse.
